# Supplementary material for: Pomegranate Extract Augments Energy Expenditure Counteracting the Metabolic Stress Associated with High-Fat-Diet-Induced Obesity
Source: Int J Mol Sci. 2022 Sep 9;23(18):10460. doi: 10.3390/ijms231810460 (PMC9499678; doi:10.3390/ijms231810460)
Supplement: Supplementary file 1 [file ijms-23-10460-s001.zip › Suppl. Material png/Supplementary Table S1.pdf]

Supplementary Table S1. Taqman probes used in the study

GENE	Probe
UCP1	Mm01244861_m1
CPT1a	Mm00550448_m1
SIRT1	Mm01168521_m1
PGC1a	Mm01208835_m1
PPARG	Mm00440940_m1
TFAM	Mm00447485_m1
PRDM16	Mm00712556_m1
BMP8B	Mm00432115_g1
SREBF1	Mm00550338_m1
AMPK	Mm01296700_m1
EPDR1	Mm07299958_m1
B2M	Mm00437762_m1
IL17RA	Mm00434214_m1
IRS1	Mm01211875_m1
PFK	Mm01309576_m1
LDH	Mm00459144_m1
G6PDH	Mm00658204_s1
