# Supplementary figures and images for: Pomegranate Extract Augments Energy Expenditure Counteracting the Metabolic Stress Associated with High-Fat-Diet-Induced Obesity
Source: Int J Mol Sci. 2022 Sep 9;23(18):10460. doi: 10.3390/ijms231810460 (PMC9499678; doi:10.3390/ijms231810460)

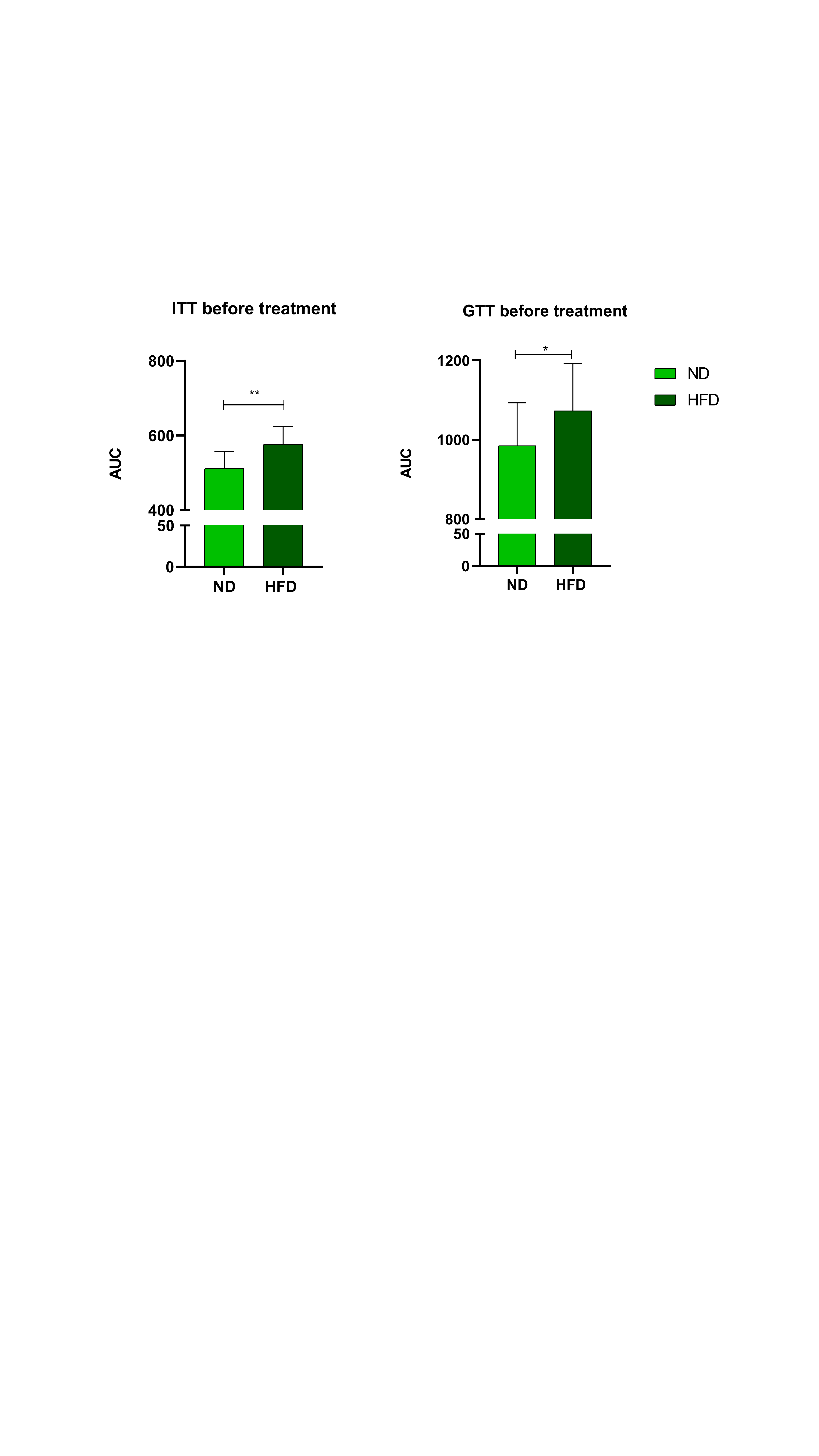

Supplement: Supplementary file 1 [file ijms-23-10460-s001.zip › Suppl. Material png/Supl Fig S1.png]

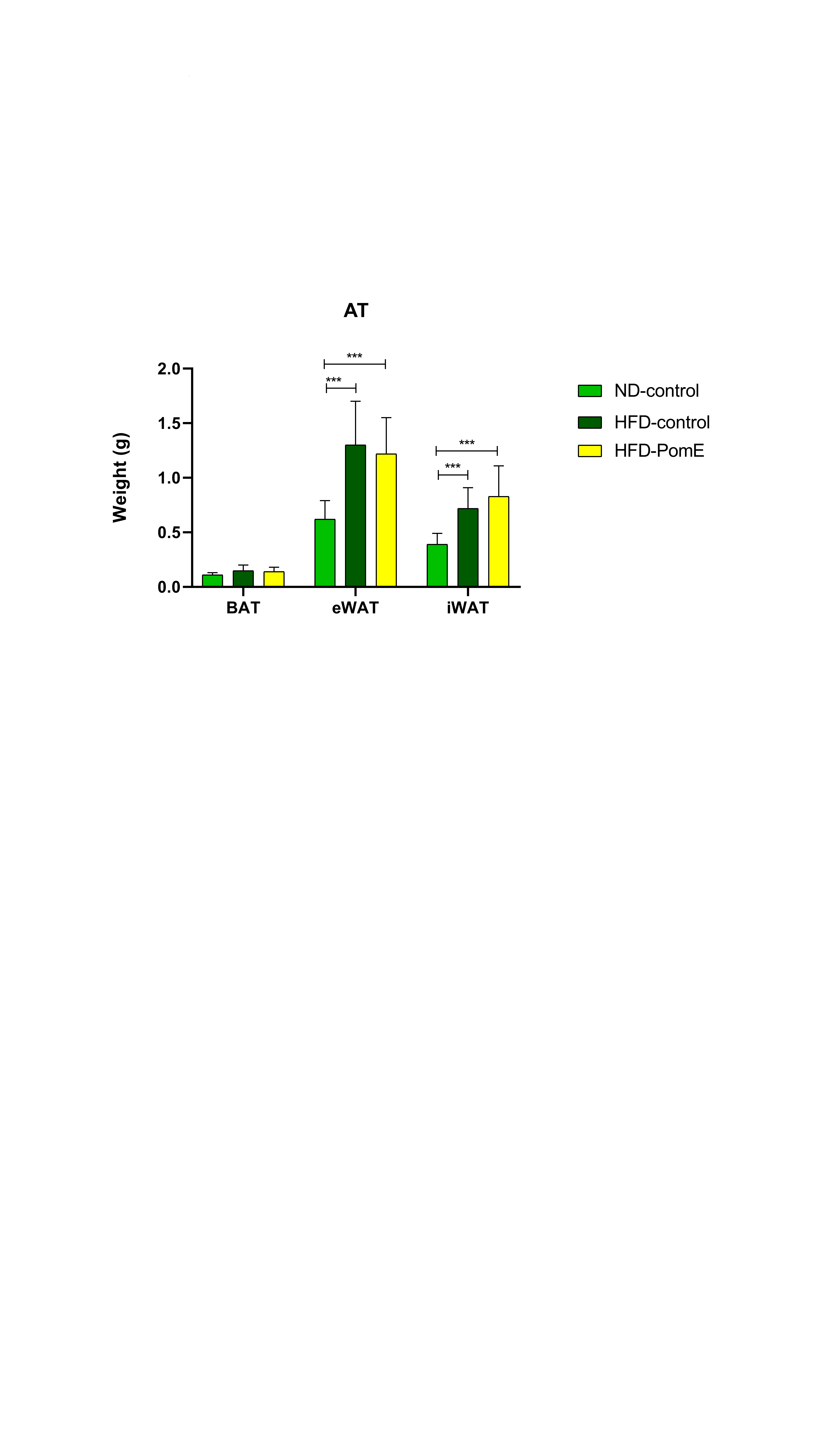

Supplement: Supplementary file 1 [file ijms-23-10460-s001.zip › Suppl. Material png/Supl. Fig S2.png]

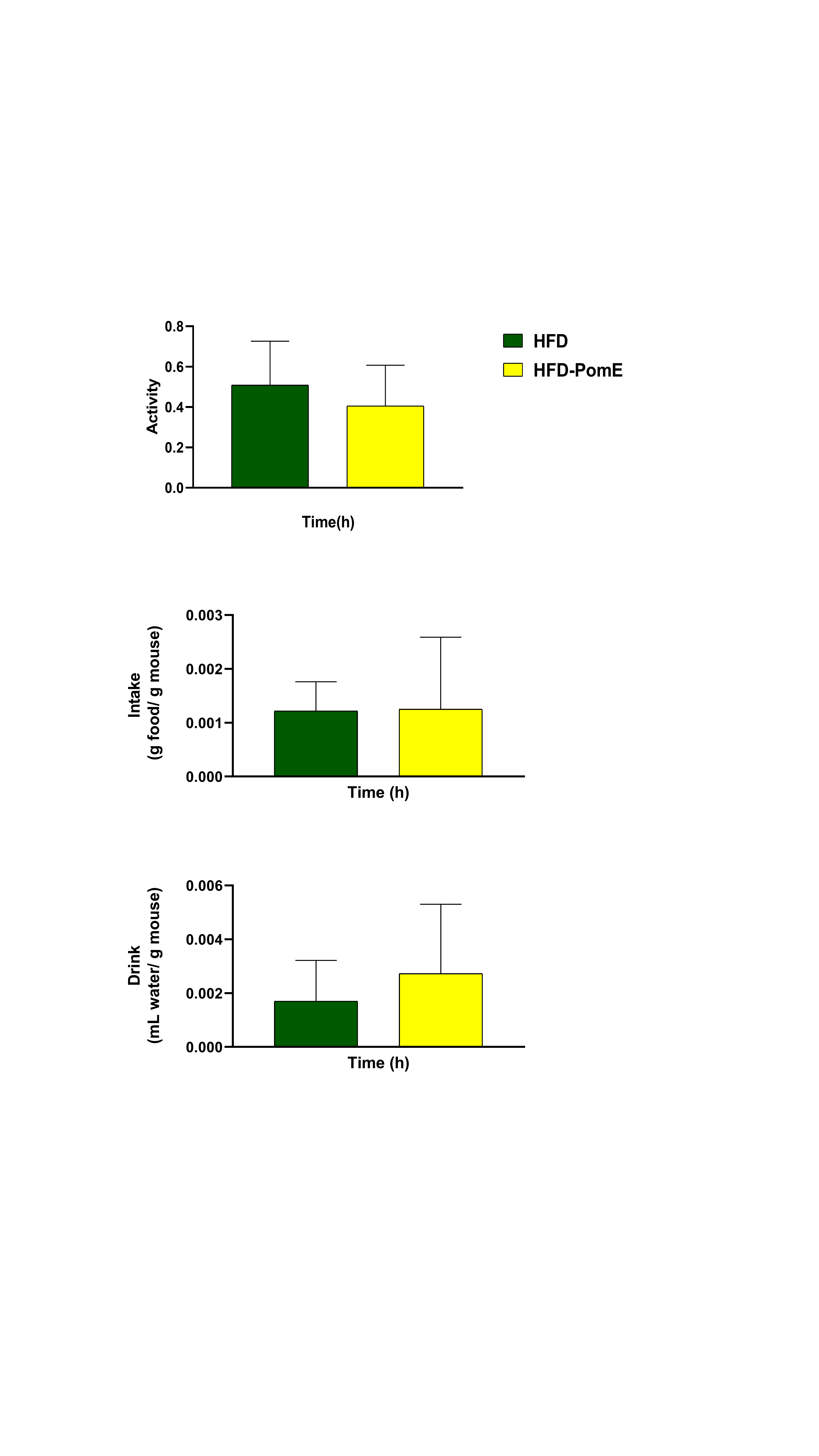

Supplement: Supplementary file 1 [file ijms-23-10460-s001.zip › Suppl. Material png/Supl. Fig S3.png]
